# Sustained speed of kill and repellency of a novel combination of fipronil and permethrin against Ctenocephalides canis flea infestations in dogs

**DOI:** 10.1186/s13071-015-0680-1

**Published:** 2015-01-27

**Authors:** Frederic Beugnet, Mark Soll, Emilie Bouhsira, Michel Franc

**Affiliations:** Merial S.A.S., 29 Av. Tony Garnier, 69007 Lyon, France; Service de Parasitologie, Ecole Nationale Vétérinaire de Toulouse, 23 Chemin des Capelles, 31076 Toulouse, France

**Keywords:** Dogs, Fleas, *Ctenocephalides canis*, Fipronil, Permethrin, Speed of kill, Repellency

## Abstract

**Background:**

*Ctenocephalides canis* is a major flea species in dogs in several European countries. The new topical combination of fipronil and permethrin (Frontline Tri-Act®/Frontect®, Merial) has been developed to control fleas, ticks, mosquitoes, sandflies and biting flies on dogs. Considering the repellent and insecticidal effects of permethrin and the insecticidal effect of fipronil, the efficacy of the combination against fleas including *C. canis* was expected to be rapid. The study was conducted to measure the 1-hour, 6-hour and 24-hour efficacy, as well as the repellent activity, of the fipronil-permethrin combination on treated *versus* untreated dogs.

**Methods:**

12 Beagle dogs were randomly allocated to one of two groups based on pre-treatment live flea counts. Dogs in Group 1 remained untreated whereas dogs in Group 2 were treated once on Day 0. Each dog was infested with 100 unfed adult *C. canis* on Days 2, 7, 14, 21 and 28. Dogs were combed for fleas 1 and 6 h after each infestation. Following this examination, fleas remaining on the liner at the bottom of each cage were collected and counted. All live fleas were placed back on each dog after the 1- and 6-hour counts. A comb-count was performed at 24 h post infestation on all dogs.

**Results:**

Treated dogs had significantly (*p* ≤ 0.01) lower flea counts than untreated dogs at every time point. The percent efficacy was ≥99.1% at 6 and 24 h after each weekly challenge up to the month. The 1-hour counts also showed good efficacy of 96.5%, 98.9%, 92.0%, 70.2% and 55.7% on Days 2, 7, 14, 21 and 28, respectively. The repellent efficacy, assessed on the liners at 1 h, was 86.5%, 94.9%, 79.5%, 58.4% and 43.9% on Days 2, 7, 14, 21 and 28, respectively.

**Conclusions:**

This study demonstrates the beneficial effect of the fipronil and permethrin combination against *C. canis*, providing both a repellent and insecticidal effect as early as 1 h post infestation, and >99.1% efficacy calculated at 6 h during a month.

## Résumé

**Contexte:**

*Ctenocephalides canis* est une espèce de puces importante chez les chiens dans plusieurs pays d’Europe. La nouvelle combinaison topique de fipronil et de perméthrine (Frontline Tri-Act®/Frontect®, Merial) a été développée pour lutter contre puces, tiques, moustiques, phlébotomes et mouches piqueuses chez le chien. Etant donné les propriétés répulsives et insecticides de la perméthrine et l’action insecticide du fipronil, l’efficacité de la combinaison contre les puces, dont *C. canis*, devrait être rapide. L’étude a été conduite afin de mesurer l’efficacité à 1 heure, 6 heures et 24 heures, mais aussi le caractère répulsif de la combinaison fipronil-perméthrine chez les chiens traités par rapport aux chiens non traités.

**Méthode:**

12 chiens de race Beagle ont été répartis de manière aléatoire dans l’un des deux groupes sur la base des résultats d’une infestation par les puces avant traitement et durant la phase d’acclimatation. Les chiens du groupe 1 ne sont pas traités alors que les chiens du groupe 2 sont traités une fois à J0. Chaque chien est infesté par 100 puces *C. canis* adultes à jeun, aux jours 2, 7, 14, 21 et 28. Les chiens sont peignés à la recherche de puces 1 et 6 heures après chaque infestation. Après cet examen, les puces qui restent sur le revêtement de sol de chaque cage sont collectées et comptées. Après les peignages-comptages à 1 heure et 6 heures, toutes les puces vivantes sont replacées sur les chiens. Un dernier comptage par peignage est réalisé pour chaque chien 24 heures après infestation.

**Résultats:**

Les chiens traités ont eu significativement moins de puces (p ≤ 0,01) que les chiens non traités, à chaque point de comptage. L’efficacité était ≥ 99,1% à 6 h et 24 h après chaque infestation hebdomadaire durant un mois. L’efficacité à 1 heure était également élevée avec 96,5,%, 98,9%, 92,0%, 70,2% et 55,7% aux jours 2, 7, 14, 21 et 28, respectivement. L’efficacité “répulsive”, évaluée sur le revêtement de sol à 1 h, était de 86,5%, 94,9%, 79,5%, 58,4% et 43,9% aux jours 2, 7, 14, 21 et 28, respectivement.

**Conclusions:**

Cette étude démontre l’effet bénéfique de la combinaison de fipronil et perméthrine contre *C. canis*, qui apporte une action à la fois insecticide et « répulsive » dès 1 h post infestation, et >99,1% d’efficacité à 6 h pendant un mois.

## Background

Although the cat flea *Ctenocephalides felis* is considered to be the predominant flea species found on both cats and dogs worldwide [1-3], the prevalence of *Ctenocephalides canis* appears to be greater than previously believed in many regions [1,4]. *C. canis* is the predominant flea species of dogs in eastern and central European countries [5-10]. Even in areas where *C. felis* appears to be predominant like in Western Europe, representing 80% or more of species identified on dogs, the prevalence of *C. canis* in dog populations may still be as high as 10-20% [11-13]. In a recent survey, Beugnet et al. found 12.5% of French flea infested dogs to be infested by *C. canis* and 21.4% of German flea infested dog to harbor *C. canis* [14].

The present study was conducted in order to determine the efficacy of a new topical spot-on combination containing both permethrin and fipronil against the dog flea, *C. canis*. Several marketed ectoparasiticides have already a known efficacy on *Ctenocephalides canis* [15] and are used regularly to control fleas on dogs [1]. This new combination is intended to control fleas, ticks, mosquitoes, sandflies and biting flies on dogs. The potential benefits of such a combination against fleas could be an insecticidal effect related to both permethrin and fipronil, which could increase the speed of kill, but also a knock-down repellent effect linked to permethrin. To demonstrate the possible speed of kill, the study design presented here assessed the flea numbers on dogs at 1 and 6 h post flea-challenge. This combination could also potentially maintain its efficacy level against less susceptible flea strains to one of its compound, a combined decrease of efficacy to two insecticides having completely different modes of action being really unlikely [1,15].

The repellent activity against fleas is controversial [16]. Repellency *sensu stricto* is well defined by a fly-away effect which is characterized in flying insects (mosquitoes, sandflies, flies). It is usually calculated by the measurement of an anti-feeding effect observed after 1 hour of exposure to treated animals [16,17]. The repellency *sensu lato* includes effects on insects or acarians infesting their hosts but leaving it quickly. It can be related to an irritant effect by contact, or a behavioral signal to prevent attachment or bite and leave. The first is described with permethrin and ticks, the second with amitraz and ticks [16]. Fleas jump to infest their host, therefore, we can hypothesize that any repellent effect will follow a contact with a treated skin. Based on what is known for ticks, the irritant effect of permethrin, probably linked with a knock-down effect, would induce fleas to fall off their host. As it has been described for ticks, the design of the study included the possibility to count the fleas on the ground plate of each dog after 1 or 6 hours of exposure, therefore allowing to estimate a percent of repellency *sensu lato* [17].

## Methods

### Study design

The study was designed in accordance with the “World Association for the Advancement of Veterinary Parasitology (W.A.A.V.P.) guidelines for evaluating the efficacy of parasiticides for the treatment, prevention and control of flea and tick infestation on dogs and cats” [17] and was conducted in accordance with Good Clinical Practices as described in International Cooperation on Harmonisation of Technical Requirements for Registration of Veterinary Medicinal Products (VICH guideline 9). All animals were managed similarly, with due regard for their well-being and in compliance with Merial Ethics Committee and applicable French regulations and requirements.

The study was a negative control, efficacy study using a randomized block design with blocks based on pre-treatment live flea counts. Each dog was an experimental unit and was treated and assessed for the study variables on an individual basis. Included dogs had not been treated with ectoparasiticides (either topical or systemic) within 3 months of the start of the study. The animals were housed individually and each group was housed separately to prevent contact between animals in the different groups.

Twelve dogs (nine females and three males) weighing 9.4 to 12.6 kg were ranked based upon their flea holding capability and allocated to the untreated control group (6 dogs) or to the Frontect® treated group (6 dogs). The dogs were treated on Day 0, and flea challenged on days 2, 7, 14, 21 and 28 (see below). They were observed hourly during 4 hours after treatment to identify any adverse events, and then they were observed daily for any clinical change.

### Treatment

The topical combination of fipronil and permethrin (Frontect®, Merial) contains 6.76% w/v fipronil and 50.48% w/v permethrin to deliver a minimum dose of 6.76 mg/kg fipronil and 50.48 mg/kg permethrin. For dogs weighing > 5 to 10 kg, 1 mL was applied, while 2 mL was applied to dogs weighing >10 to 20 kg. The product was applied as directed on the label, in two spots, on the back at the base of the neck in front of the shoulder blades and on the middle of the neck between the base of the skull and the shoulder blades. Application at these sites prevents oral ingestion of the product.

### Parasites

A wild strain of *C. canis* isolated in 2008 from hunting dogs in the area of Montesquieu-Volvestre (Haute-Garonne, France) was used. The strain has been maintained under laboratory conditions since that time. The strain is not known to be resistant to known ectoparasiticide.

### Flea infestations and counts

To assess efficacy, the 12 dogs were infested on Days 2, 7, 14, 21 and 28. After each flea infestation, the dogs were comb-examined to collect and count live, moribund and dead fleas at 1 h post-infestation (PI), and then again at 6 h and 24 h. At 1 h and 6 h, the live fleas were placed back on their dog.

Dead fleas are easily identified because they are immobile. The moribund fleas were defined as dying fleas, which where trembling, convulsing and were not able to move normally. Moribund fleas were added to the dead fleas for the evaluation of efficacy, which takes into account the number of live fleas on dogs, following the WAAVP as well as registration agencies guidelines [17].

To assess the repellency *sensu lato*, live, moribund and dead fleas were also collected on the liners in each cage at 1 h PI and 6 h PI.

### Data analysis

Insecticidal efficacy (based on live flea counts) was calculated at 1, 6 and 24 h PI using the Abbott’s formula, as recommended by WAAVP guidelines [17]. Both arithmetic and geometric means were calculated, but the Geo means giving a better central tendency, they were used to calculate the % of efficacy. Counts of live adult fleas were transformed to the natural logarithm of (count + 1) for calculation of geometric means by treatment group at each time point. Percent reduction from the corresponding control mean was calculated using the formula: % efficacy = [(C – T) / C]*100, where C = geometric mean for the control group, and T = geometric mean for the treated group.

Repellent efficacy (% repellency) was calculated at 1 h PI based on numbers of fleas collected on the liner, whatever their status (dead, moribund or live). Percent repellency was calculated using the formula: % repellency = [(L / F)*100], where L = number of fleas collected on the liner at 1 h PI, and F = total number of fleas collected from the liner and the dogs.

Based on the fact that the number of dogs was the same in each group and that each dog was infested by 100 fleas, the corrected repellent efficacy (% corrected repellency) was calculated using the formula: % corrected repellency = % repellency_TG_ – % repellency_CG_, where % repellency_TG_ = % repellency observed in Treated Group (TG) and % repellency_CG _= % repellency observed in the Control Group (CG).

Statistical analysis used the Wilcoxon rank sum test with continuity correction. The residues of the model cannot have a normal distribution, mainly because there is no variation in the data from Control Group. The statistical analysis was performed using R language [18].

## Results

Dogs administered a single topical treatment with Frontect® had significantly (*p* ≤ 0.01) lower flea counts than untreated controls at every counting time point (i.e., 1, 6 and 24 h). The percent insecticidal efficacy, as assessed by live flea counts on dogs, was ≥99.1% at 6 and 24 h through the last infestation on Day 28 (Table 1). The 1-hour counts also showed good efficacy of 96.5%, 98.9%, 92.0%, 70.2% and 55.7% on Days 2, 7, 14, 21 and 28, respectively.

**Table 1:** **Efficacy of a fipronil-permethrin combination (Frontect®) against** ***Ctenocephalides canis*** **flea challenges in dogs**

**Counts on dogs at 1 h post-infestation (PI)**						
**Groups**		**Day 2**	**Day 7**	**Day 14**	**Day 21**	**Day 28**
**Treated (6 dogs)**	GM	2.97	0.98	7.09	23.89	39.08
**Control (6 dogs)**	GM	84.98	88.99	89.12	80.23	88.26
	*p *value	0.004922	0.004772	0.004998	0.004998	0.01027
	**%Efficacy**	**96.5**	**98.9**	**92.0**	**70.2**	**55.7**
**Counts on dogs at 6 h PI**						
		Day 2	Day 7	Day 14	Day 21	Day 28
**Treated**	GM	0.00	0.00	0.00	0.00	0.74
**Control**	GM	73.22	81.64	81.01	68.10	79.91
	*p *value	0.002725	0.002778	0.002778	0.002725	0.004698
	**%Efficacy**	**100.0**	**100.0**	**100.0**	**100.0**	**99.1**
**Counts on dogs at 24 h PI**						
**Treated**	GM	0.00	0.00	0.00	0.00	0.00
**Control**	GM	66.76	77.63	73.61	64.32	76.30
	*p *value	0.002778	0.002725	0.002778	0.002778	0.002778
	**%Efficacy**	**100.0**	**100.0**	**100.0**	**100.0**	**100.0**

GM = geometric mean. Table lines in Bold = Result lines highlighted.

The corrected repellency observed at 1 h PI was 86.5%, 94.9%, 79.5%, 58.4% and 43.9% on Days 2, 7, 14, 21 and 28, respectively (Table 2).

**Table 2:** **Repellent efficacy of a fipronil-permethrin combination against** ***Ctenocephalides canis*** **determined by numbers of fleas removed from the cage liner at 1 h post-infestation of dogs**

		**Day 2**	**Day 7**	**Day 14**	**Day 21**	**Day 28**
Treated Group (6 dogs)	Total number of fleas (collected on dogs + liners)	525	529	436	482	511
	Total number of fleas collected on liner (dead)	472 (427)	501 (498)	359 (313)	291 (150)	231 (191)
	% Repellency	89.9%	94.7%	82.3%	60.4%	42.2%
Control Group (6 dogs)	Total number of fleas (collected on dogs + liners)	534	540	556	502	540
	Total number of fleas collected on liners (dead)	18 (18)	4 (4)	16 (16)	10 (10)	7 (7)
	% Repellency	3.4%	0.7%	2.9%	2.0%	1.3%
% Corrected repellency at 1 h PI*		**86.5%**	**94.0%**	**79.5%**	**58.4%**	**43.9%**

*PI: Post Infestation. Table lines in Bold = Result lines highlighted.

A significant repellency effect (*p* < 0.05) was achieved by 1 h post-infestation for a full month. It was linked with an important mortality, ranging from 82.7% to 99%, of these repelled fleas.

One control dog demonstrated erythema and alopecia on the back of the tail between Days 3 and 16 due to flea allergy and was treated topically with chlorhexidine spray. No adverse events were observed following treatment.

## Discussion

Treatment of dogs with the new fipronil-permethrin topical combination provided effective control (≥99%) of the dog flea for 4 weeks after treatment. This rate of control is above the threshold requested to get the indication on the label. Overall, efficacy is similar to that obtained on *C. canis* with fipronil, where 99.6% and 100% efficacy was observed for 37 days after treatment [4,19]. Spinosad administered orally following United States labeling (at doses ranging from 31.65 to 54.85 mg/kg) has been shown to have good 24-hour efficacy for 3 weeks only [20].

More interesting, the efficacy > 99% was seen as early as 6 h post-infestation, which is earlier than the conventional timing of flea counts done at 24 or even at 48 h. The results of the current study highlight the speed of kill provided by the combination of the two active ingredients. In the present study, the insecticidal efficacy was ≥ 96.5% when evaluated 1 h after infestation during the first week, and 92% up to and including Day 14. Such a significant start of kill at 1 h and almost complete at 6 h, during a month, is most probably related to an added effect of both permethrin and fipronil acting together on different targets. The high efficacy observed on Day 2 following treatment on Day 0 is an indication that both active ingredients translate across the skin of the dogs within 24 h.

In addition, repellency *sensu lato* was significant for the full month with a peak of 94.0% observed on Day 7. This repellent effect is most probably due to the known mode of action of permethrin [15]. The definition and calculation of repellency is sometimes controversial [15,16]. It is commonly assessed on mosquitoes, sandflies or biting flies by the evaluation of the feeding rate at 1 h after exposure of dogs under a net (VICH Guideline 9). It could be evaluated for fleas or ticks by counting dead and live arthropods on the animal compared to the control, when efficacy is only based on live counts, or it can be evaluated by the numbers of arthropods found off the host. In the latter case, the design should allow the collection of fleas/ticks out of the animals. It cannot be performed a long time after exposure due to the risk to lose the fleas or ticks, which can be scratched or ingested by the animals, defiled urine or feces, or simply disappeared. The design of this study allowed the collection of fleas in the liner of the cage of each dog, but due to this risk of bias, the authors considered that the evaluation at 1 h PI was better than at 6 h PI. The design of the study allowed confirmation that repellency *sensu lato* can be assessed on fleas and does exist at a significant level. The repellency is linked with a mortality rate which was not negligible. In the treated group, compared to the control one, the majority of fleas that fell off the dogs were already dead (82.7 to 99%), but not all, demonstrating a combination of both knock-down and insecticidal effects (Table 2). This mortality rate is of epidemiological importance as the repelled fleas will not be able to re-infest their host.

## Conclusions

This study demonstrates the beneficial effect of the fipronil and permethrin combination against the dog flea, providing both a repellent and insecticidal effect as early as 1 h post infestation for a full month, and a speed of kill above 99% at 6 h during a month. The benefit of the repellency and speed of kill in the control of fleas will be obvious by a quick visible effect for both the veterinarians and the dog owners.

## References

[1] Halos L, Beugnet F, Cardoso L, Farkas R, Franc M, Guillot J (2014). Flea control failure? Myths and realities. Trends Parasitol.

[2] Rust MK, Dryden MW (1997). The biology, ecology, and management of the cat flea. Annu Rev Entomol.

[3] Traversa D (2013). Fleas infesting pets in the era of emerging extra-intestinal nematodes. Parasit Vectors.

[4] Bouhsira E, Yoon SS, Roques M, Manavella C, Vermot S, Cramer LG (2011). Efficacy of fipronil, amitraz and (S)-methoprene combination spot-on for dogs against adult dog fleas (*Ctenocephalidescanis*, Curtis, 1826). Vet Parasitol.

[5] Koutinas AF, Papazahariadou MG, Rallis TS, Tzivara NH, Himonas CA (1995). Flea species from dogs and cats in northern Greece: environmental and clinical implications. Vet Parasitol.

[6] Xhaxhiu D, Kusi I, Rapti D, Visser M, Knaus M, Lindner T (2009). Ectoparasites of dogs and cats in Albania. Parasitol Res.

[7] Farkas R, Gyurkovszky M, Solymosi N, Beugnet F (2009). Prevalence of flea infestation in dogs and cats in Hungary combined with a survey of owner awareness. Med Vet Entomol.

[8] Chee JH, Kwon JK, Cho KO, Lee YJ, Abdel-Aty AM, Shin SS (2008). A survey of ectoparasite infestations in stray dogs of Gwang-ju City, Republic of Korea. Korean J Parasitol.

[9] Guzman RF (1984). A survey of cats and dogs for fleas; with particular reference to their role as intermediate hosts of *Dipylidiumcaninum*. NZ Vet J.

[10] Gonzales A, Castro DC, Gonzales S (2004). Ectoparasitic species from *Canis familiaris* (Linné) in Buenos Aires province, Argentina. Vet Parasitol.

[11] Franc M, Choquart P, Cadiergues MC (1998). Répartition des espèces de puces rencontrées chez le chien en France. Rev Med Vet.

[12] Gracia MJ, Calvete C, Estrada R, Castillo JA, Peribanez MA, Lucientes J (2007). Fleas parasiting domestic dogs in Spain. Vet Parasitol.

[13] Beck W, Boch K, Mackensen H, Wiegand B, Pfister K (2006). Qualitative and quantitative observations on the flea population dynamics of dogs and cats in several areas of Germany. Vet Parasitol.

[14] Beugnet F, Labuschagne M, Fourie J, Guillot J, Farkas R, Cozma V (2014). Occurrence of *Dipylidium caninum* in fleas from client-owned cats and dogs in Europe using a new PCR detection assay. Vet Parasitol.

[15] Beugnet F, Franc M (2012). Insecticide and acaricide molecules and/or combinations to prevent pet infestation by ectoparasites. Trends Parasitol.

[16] Halos L, Baneth G, Beugnet F,Bowman AS, Chomel B, Farkas R, Franc M, Guillot J, Inokuma H, Kaufman R, Jongejan F, Joachim A, Otranto D, Pfister K, Pollmeier M,Sainz A, Wall R: Defining the concept of “tick repellency” in veterinary medicine. Parasitology. 2012;139(4):419–423. doi: 10.1017/S0031182011002228 10.1017/S0031182011002228 PMC3302427 22216951

[17] Marchiondo AA, Holdsworth PA, Fourie LJ, Rugg D, Kellmann K, Snyder DE (2013). World Association for the Advancement of Veterinary Parasitology (W.A.A.V.P.) second edition: guidelines for evaluating the efficacy of parasiticides for the treatment, prevention and control of flea and tick infestations on dogs and cats. Vet Parasitol.

[18] R: A language and environment for statistical computing. R Foundation for Statistical Computing, Vienna, Austria. ISBN 3-900051-07-0, URL http://www.R-project.org).

[19] Cadiergues MC, Caubet C, Franc M (2001). Comparison of the activity of selamectin, imidacloprid and fipronil for the treatment of dogs infested experimentally with *Ctenocephalides canis* and *Ctenocephalides felis felis*. Vet Rec.

[20] Franc M, Bouhsira E (2009). Evaluation of speed and duration of efficacy of spinosad tablets for treatment and control of *Ctenocephalides canis* (Siphonaptera: Pulicidae) infestations in dogs. Parasite.

